# Anti-NR2A/B Antibodies and Other Major Molecular Mechanisms in the Pathogenesis of Cognitive Dysfunction in Systemic Lupus Erythematosus

**DOI:** 10.3390/ijms160510281

**Published:** 2015-05-06

**Authors:** Sen Hee Tay, Anselm Mak

**Affiliations:** 1 Department of Medicine, Yong Loo Lin School of Medicine, National University of Singapore, 21 Lower Kent Ridge Road, Singapore 119077, Singapore; E-Mail: mdcam@nus.edu.sg; 2 Divison of Rheumatology, Department of Medicine, National University Hospital, National University Health System, 1E Kent Ridge Road, Singapore 119228, Singapore

**Keywords:** cognitive dysfunction, anti-NR2A/B antibodies, matrix metalloproteinase-9, neutrophil extracellular traps, pro-inflammatory mediators, systemic lupus erythematosus

## Abstract

Systemic lupus erythematosus (SLE) is an autoimmune disease that affects approximately 1–45.3 per 100,000 people worldwide. Although deaths as a result of active and renal diseases have been substantially declining amongst SLE patients, disease involving the central nervous system (CNS), collectively termed neuropsychiatric systemic lupus erythematosus (NPSLE), remains one of the important causes of death in these patients. Cognitive dysfunction is one of the most common manifestations of NPSLE, which comprises deficits in information-processing speed, attention and executive function, in conjunction with preservation of speech. Albeit a prevalent manifestation of NPSLE, the pathogenetic mechanisms of cognitive dysfunction remain unclear. Recent advances in genetic studies, molecular techniques, neuropathology, neuroimaging and cognitive science have gleaned valuable insights into the pathophysiology of lupus-related cognitive dysfunction. In recent years, a role for autoantibodies, molecular and cellular mechanisms in cognitive dysfunction, has been emerging, challenging our previous concept of the brain as an immune privileged site. This review will focus on the potential pathogenic factors involved in NPSLE, including anti-*N*-methyl-d-aspartate receptor subunit NR2A/B (anti-NR2A/B) antibodies, matrix metalloproteinase-9, neutrophil extracellular traps and pro-inflammatory mediators. Better understanding of these mechanistic processes will enhance identification of new therapeutic modalities to halt the progression of cognitive decline in SLE patients.

## 1. Introduction

Systemic lupus erythematosus (SLE) is a multi-systemic autoimmune disease of unknown etiology with heterogeneous clinical manifestations, including diverse neuropsychiatric manifestations [1]. SLE affects approximately 1–45.3 per 100,000 people worldwide, with up to 90% of the cases occurring in women of childbearing age [2,3,4]. Nervous system disease in SLE includes neurologic and psychiatric events, of which 19%–38% is attributable to SLE [5]. As patients with SLE are living longer, NPSLE has been identified as one of the important factors that hampering the improvement of survival of SLE patients over the past 50 years [6,7]. NPSLE negatively impacts on health-related quality of life and has been linked to increased unemployment rates [8,9,10]. Multiple neuropsychiatric events during the disease course are associated with adverse long-term prognosis with an increased mortality rate of 7%–19% [8]. Clinical manifestations of NPSLE range from overt neurologic or psychiatric dysfunction, such as stroke or psychosis, to more subtle and subclinical abnormalities such as cognitive dysfunction, headache and mood disorders [11]. Around 50%–60% of NPSLE events occur at the onset of SLE or within the first year of diagnosis, commonly in the presence of generalized disease activity [12]. However, it has also become clear that many of the common manifestations of NPSLE are not associated with metrics of diseases, such as flare or severity [6]. NPSLE is difficult to diagnose, secondary factors such as drugs, metabolic abnormalities or infections can contribute to neuropsychiatric disturbances in SLE patients and need to be meticulously excluded [8]. NPSLE is challenging to treat with a lack of controlled randomized trials; the current therapeutic approach to NPSLE remains largely empirical [11]. Thus, there is a need to explore new paradigms for pathophysiologic mechanisms to explain and treat this increasingly important and vexing problem in SLE [6].

Cognition is the sum of intellectual functions resulting in thought and executive function, which comprises reception of external stimuli, information processing, learning, storage and expression [13]. Disturbance of even one of these functions can result in disruption of normal thought [13]. Cognitive dysfunction is a distinct subset of NPSLE and is the most prevalent manifestation of NPSLE [14,15,16]. During the 1980s, Denburg first established the presence of cognitive dysfunction in SLE patients using formal neuropsychological testing [17,18]. Cognitive dysfunction has been subsequently reported in 20%–80% of SLE patients, depending on the methodological and diagnostic practices used [19,20]. Using the one-hour neuropsychological test battery proposed by the American College of Rheumatology in 1999, in various modifications, 15%–80% of SLE patients were diagnosed to have cognitive dysfunction, thus yielding similar results compared to earlier studies [20,21]. Commonly reported domains of impairment include attention, verbal and visual learning and memory, visuospatial constructional skills, cognitive flexibility, executive functioning and psychomotor speed [22,23]. Dominant patterns of cognitive deficits have not emerged although the various patterns of impairments suggest a subcortical syndrome reminiscent of white matter dementias [21,22,24]. The course of cognitive dysfunction in SLE has been described as evanescent and fluctuating over a 2–5 year period [22,24]. However, cognitive impairment may be progressive in nature, sometimes reaching the severity of dementia in 3%–5% of lupus patients [12,22,23]. Signs of cognitive dysfunction in SLE are often subtle and difficult to ascertain clinically [25]. Relying on patient reporting of cognitive status may not be sufficient given discrepancies between subjective reporting of cognitive difficulties and objective findings on cognitive testing [22]. Stratifying patient groups by patterns of dysfunction or in the presence of various risk factors (e.g., anti-NR2A/B antibodies) may help to identify subgroups with specific underlying pathogenesis [24].

Conventional magnetic resonance imaging (MRI) remains the gold standard imaging technique for the diagnosis of NPSLE [26]. Cross-sectional and longitudinal studies have revealed that T2-weighted hyperintensities and cerebral atrophy are the most common abnormalities associated with cognitive dysfunction [12,27,28,29]. These T2-weighted hyperintensities are small punctuate lesions localized mainly to the periventricular and subcortical white matter, suggesting that white matter tract damage might be a potential mechanism for cognitive dysfunction [24,27]. SLE patients with no focal neurological symptoms who underwent ^18^F-fluorodeoxyglucose (^18^FDG) positron emission tomography (PET) had increased ^18^FDG uptake in the white matter, indicative of inflammatory activity [30]. The exact cause of cerebral atrophy is not clear but SLE patients with severe cognitive impairment had greater white and grey matter reduction compared to patients with moderate or no cognitive impairment [28]. One potential explanation of grey matter disease is a form of disconnection-induced diaschisis, in which diffuse loss of white matter structural integrity causes cortical functional decline in remote regions [30]. Hence, cognitive dysfunction in SLE may result from white matter inflammation as an inciting pathology and T2-weighted hyperintensities and cerebral atrophy develop over time [30].

In this review we will discuss the recent advances in cognitive dysfunction research from murine models and human SLE where a role for autoantibodies, molecular and cellular mechanisms has emerged. The choice of mechanisms considered in this review has been driven by earlier, albeit controversial, reports from the medical literature [16]. However, affirmatively, a recent study screening a multimarker panel for cognitive dysfunction in childhood-onset SLE identified anti-NR2A/B antibodies and elements of blood-brain barrier (BBB) disruption and neutrophil activation as potential biomarkers [31]. Hence, key to this discussion is the idea that when considered individually, the impact of these mechanisms might be muted but they likely function together, as an ensemble, to contribute to disease pathogenesis of lupus-related cognitive dysfunction.

## 2. Mechanisms of Cognitive Dysfunction

### 2.1. Anti-N-methyl-d-aspartate Receptor Subunit NR2A/B Antibodies

Anti-*N*-methyl-d-aspartate (NMDA) receptor subunit NR2A/B antibodies have been demonstrated in the sera of 30%–40% of SLE patients [32]. It appears to be a convincing candidate to mediate cognitive dysfunction in SLE patients, fulfilling four of the six stringent pathogenicity criteria outlined for autoantibodies [33]. In the pioneering work by Diamond which aimed to determine cross-reactive antigens for anti-double stranded deoxyribonucleic acid (anti-dsDNA) antibodies, the nephritogenic mouse monoclonal anti-dsDNA antibody (R4A antibody) was reacted with a phage peptide display library harboring random 10 amino acid inserts encoded by random 30-base pair insets [34,35]. A consensus sequence for the R4A antibody, D/E-W-D/E-Y-S/G, was identified in 36 phage clones [34]. Of these, 23 phage clones had identical DWEYSVWLSN amino acid sequences [34]. A protein database search found that the D/E-W-D/E-Y-S/G consensus sequence was found in some bacterial antigens but more surprisingly, also present in the *N*-terminal domains of mouse, rat and human NR2A and NR2B subunits but not in NR2C and NR2D subunits [35,36,37,38]. The *N*-terminal domains of NR2A and NR2B subunits are composed of approximately the first 350 amino acids of the protein [39]. The D/E-W-D/E-Y-S/G consensus sequence is localized to residues 283–287 (sequence DWDYS) of the NR2A subunit and residues 284–288 (sequence EWDYG) of the NR2B subunit [2].

The evidence that R4A antibody cross-reacts with NR2A and NR2B subunits of NMDA receptors prompted researchers to search for similar antigenic specificities in the polyclonal anti-dsDNA antibodies of SLE patients. Diamond demonstrated that anti-NR2A/B antibodies isolated from the sera of four SLE patients, using a DWEYSVWLSN peptide-conjugated sepharose column, cross-reacted with dsDNA on ELISA [36]. Yoshio demonstrated that affinity-purified anti-NR2A/B antibodies from 14 SLE patients cross-reacted with dsDNA in a dose-dependent manner [40]. Using an octameric DWEYSVWLSN peptide for inhibition ELISA experiments, 20%–75% inhibition was observed when the sera of 10 SLE patients were investigated for inhibition of binding of anti-dsDNA antibodies to dsDNA [41]. *In vitro* and *in vivo* experiments, using affinity-purified anti-NR2A/B antibodies, revealed that (i) adding anti-NR2A/B antibodies to neuronal cultures caused apoptotic cell death; (ii) injecting anti-NR2A/B antibodies sterotaxically into C57BL/6 mice hippocampus caused neuronal loss in the hippocampus; and (iii) intravenous administration of anti-NR2A/B antibodies into BALB/c mice with LPS treatment led to binding of these antibodies to the hippocampal neurons and caused neuronal damage [36,38]. In addition, IgG eluted from the brain of a SLE patient who had progressive and profound cognitive impairment showed cross-reactivity to dsDNA and DWEYS peptide on ELISA and mediated hippocampal neuronal damage when injected sterotaxically into a BALB/c mouse hippocampus [38]. Anti-NR2A/B antibodies from 14 SLE patients, affinity-purified using a DWEYSVWLSN peptide-conjugated sepharose column, up-regulated the expression of endothelial leukocyte adhesion molecule 1, vascular cell adhesion molecule 1 and intercellular adhesion molecule 1 on endothelial cells via the activation of NF-κB signaling pathway [40]. Expression of these endothelial cell adhesion molecules mirrored the effects of interleukin (IL)-1β in a time course experiment [40]. Several studies have indicated the presence and functionality of the NMDA receptors on brain microvascular endothelial cells (BMECs) of the BBB, suggesting the possibility of anti-NR2A/B antibodies activating BMECs through NMDA receptors [40,42]. The concentration of anti-NR2A/B antibodies measured in the CSF of 32 SLE patients with NPSLE ranged from 10 μg/mL to higher than 300 μg/mL [2]. This might imply that low titers of anti-NR2A/B antibodies in the CSF cause synaptic alteration with transient dysfunction (*i.e.*, activity), while high titers result in mitochondrial stress, causing apoptosis (*i.e.*, damage) [2].

As of April 2015, eight human studies with specific analyses performed for associations between *a priori* defined cognitive dysfunction as a NPSLE manifestation and serum anti-NR2A/B antibodies were published [9,19,31,43,44,45,46,47]. Table 1 summarizes the characteristics and findings of the studies. All eight studies synthesized DWEYSVWLSN or DWEYS peptides for ELISA testing and reported presence of anti-NR2A/B antibodies in comparison to the optical density values of the controls, each using slightly different definitions and cut-offs [9,19,31,43,44,45,46,47]. Six of the studies were cross-sectional and two studies were longitudinal [9,19,31,43,44,45,46,47]. Between 14% and 35% of the SLE patients were anti-NR2A/B antibody positive [9,19,43,44,45,46,47]. A cross-sectional study by Omdal demonstrated an association with anti-NR2A/B antibodies and cognitive impairment in 7 out of the 31 neuropsychological tests in 57 SLE patients [43]. The cross-sectional study by Massardo showed an association with anti-NR2A/B antibodies and impaired attention and executive function assessed using a computerized system in 133 women with SLE [47]. In a longitudinal study by Hanly, anti-NR2A/B antibodies levels fluctuated over time and some patients had persistently elevated levels; there was no association between a rise in or persistently elevated anti-NR2A/B antibody levels and change in cognitive function in 65 female SLE patients over a follow-up period of five years [44]. However, the longitudinal study by Brunner revealed an association between decline in working memory and an increase in anti-NR2A/B antibodies from baseline in pediatric SLE patients followed up for 18 months [31]. Studies with other *a priori* defined NPSLE manifestations have also yielded inconsistent results in correlating serum levels of anti-NR2A/B antibodies [6]. For example, two studies demonstrated an association with mood disorder (depressed mood measured using Beck Depression Inventory) and serum anti-NR2A/B antibodies, but four other studies found no such correlation [9,19,43,45,46,48]. In contrast, an association with diffuse and central NPSLE manifestations has been demonstrated in all four studies in which CSF anti-NR2A/B antibodies were measured [49,50,51,52]. Levels of CSF anti-NR2A/B antibodies were elevated in patients with diffuse and central manifestations of NPSLE compared to controls [49,50,51,52]. Titers of CSF anti-NR2A/B antibodies correlated with the severity of NPSLE manifestations [51,52]. CSF anti-NR2A/B titers were highest in SLE patients with acute confusional state (the severest form of diffuse NPSLE), followed by SLE patients with other diffuse and central NPSLE manifestations (including cognitive dysfunction and mood disorder) and lastly NPSLE manifestations involving the peripheral nervous system [52]. Q albumin, an indicator of BBB dysfunction, was highest in SLE patients with acute confusional state, followed by other diffuse and central NPSLE manifestations and lastly NPSLE manifestations involving the peripheral nervous system [52]. Further, CSF anti-NR2A/B titers correlated with Q albumin, indicating that BBB dysfunction plays an important role in the pathogenesis of NPSLE, allowing the entry of greater amounts of anti-NR2A/B antibodies from the systemic circulation into the CNS in the more severe forms of NPSLE [52]. Up to 8% of the healthy controls were classified as serum anti-NR2A/B antibody positive in four cross-sectional studies [43,46,50,53]. Interestingly, serum anti-NR2A/B antibodies have been demonstrated in patients with transient ischemic attacks and strokes using peptide-based ELISA [54,55]. Peptide fragments of NR2A and NR2B subunits have been observed in plasma of patients with acute cerebral ischemia [54]. A 21 amino acid sequence (NGMIGEVVYQRAVMAVGSLTI) was identified using a phage peptide display library to screen antibodies from patients suffering from acute ischemic stroke [54]. This amino acid sequence corresponds to residues 494–514 of the NR2A subunit and bears minor differences to the NR2B subunit [54]. These data suggest that mechanisms for production of anti-NR2A/B antibodies may be operational even in non-autoimmune healthy controls, albeit with different fine antibody specificities.

**Table 1:** Human studies on the association between anti-NR2A/B antibodies and cognitive dysfunction in systemic lupus erythematosus (SLE) patients.

References	Study Type	Number of Subjects	Amino Acid Sequence Used for Immunoassays	Reported Anti-NR2A/B Values	Essential Findings
[9]	Cross-sectional	412 SLE patients	DWEYSVWLSN	Serum optical densities; 19% of SLE patients were anti-NR2/B antibodies positive	No association between anti-NR2A/B antibody status and cognitive impairment
[19]	Cross-sectional	60 SLE patients	DWEYS	Serum optical densities; 33.3% of SLE patients were anti-NR2/B antibodies positive	No association between anti-NR2A/B antibody status and cognitive impairment
[31]	Longitudinal (18 months)	40 pediatric SLE patients	DWEYSVWLSN	Serum concentration (U/mL)	Association between decline in working memory and an increase in anti-NR2A/B antibodies from baseline
[43]	Cross-sectional	57 SLE patients	DWEYSVWLSN	Serum optical densities; 19% of SLE patients were anti-NR2/B antibodies positive	7 of the 31 neuropsychological tests associated with positive anti-NR2A/B antibodies
[44]	Longitudinal (5 years)	65 women with SLE	DWEYS	Serum optical densities; 35% of SLE patients were anti-NR2/B antibodies positive	No association between anti-NR2A/B antibody status and cognitive impairment; No association between rise in or persistently elevated anti-NR2A/B antibodies and cognitive function over 5 years
[45]	Cross-sectional	93 SLE patients	DWEYSVWLSN	Serum optical densities; 25.8% of SLE patients were anti-NR2/B antibodies positive	No association between anti-NR2A/B antibody status and cognitive impairment
[46]	Cross-sectional	43 SLE patients and 27 healthy controls	DWEYSVWLSN	Serum optical densities; 14% of SLE patients and 7.4% of healthy controls were anti-NR2/B antibodies positive	No association between anti-NR2A/B antibody status and cognitive impairment
[47]	Cross-sectional	133 women with SLE	DWEYSVWLSN	Not available	Association with impaired performance in attention and executive function with positive anti-NR2A/B antibodies

### 2.2. Matrix Metalloproteinase-9

Matrix metalloproteinases (MMPs) are a family of zinc- and calcium-dependent endoproteinases that mediate degradation and remodeling of the extracellular matrix proteins [56,57]. MMPs are secreted as inactive proenzymes and are activated extracellularly by various mechanisms [58]. The entire process is precisely regulated at the level of gene transcription, activation of the precursor proenzymes and inhibition by their endogenous inhibitors, tissue inhibitors of metalloproteinases (TIMPs) [59]. In the immune system, MMP-9 is secreted by several immunocompetent cell types (granulocytes, lymphocytes and monocytes), with neutrophils being one of the richest cellular sources of MMP-9 [58,60]. There is evidence in human and animal stroke models that activation of MMPs, specifically MMP-9, can degrade components of the basal lamina such as type IV collagen, fibronectin and laminin and contribute to proteolysis of the basal lamina and subsequent disruption of the BBB [61,62]. MMP-9 is inhibited by TIMP-1, a glycoprotein that forms a complex with MMP-9 to inhibit its proteolytic activity [63]. *Ex vivo* analyses of autopsied human brain tissue showed the following: (i) increased in total MMP-9 (pro-MMP9 and active MMP-9) in the microvessels; (ii) majority of the infiltrated cells surrounding the microvessels being MMP-9^+^ neutrophils; and (iii) type IV collagen degradation colocalizing with MMP-9^+^ neutrophils within areas of hemorrhagic and non-hemorrhagic infarcted brain tissue [62]. Neutrophil depletion in a rat model of intracerebral hemorrhage demonstrated reduced MMP-9 expression and BBB breakdown [60]. Baseline MMP-9 and MMP-9/TIMP-1 ratio have been shown to be predictive of hyperintense acute reperfusion injury, a neuroimaging marker of BBB disruption in humans, at 24 h [61]. Further, in a cross-sectional study of patients with neuromyelitis optica and multiple sclerosis, serum MMP-9 levels correlated with Q albumin and the Kurtzke’s Expanded Disability Status Scale score [57]. Hence, there is evidence that MMP-9 mediates BBB disruption and it may play a role in the neuroinflammatory process of CNS diseases.

Recent evidence has implicated MMP-9 in the pathogenesis of a variety of autoimmune diseases [64]. Peripheral blood mononuclear cells (PBMCs) from SLE patients have been shown to express higher levels of MMP-9 mRNA and pro-MMP-9 protein compared to healthy controls [65]. However, data from cross-sectional studies evaluating serum MMP-9 levels in SLE patients has been heterogeneous. Total serum MMP-9 levels were higher in SLE patients in one study, lower in SLE patients in two studies and similar to healthy controls in another study [63,64,66,67]. The activity of serum MMP-9 was higher compared to healthy controls in one study but lower in another [63,68]. These discrepancies could be due to different techniques employed to measure MMP-9 levels and activity or the presence of MMP-9 promoter polymorphisms in SLE patients [67]. In contrast, serum and CSF MMP-9 levels have been consistently shown to be elevated in patients with NPSLE. In a cross-sectional study evaluating SLE patients with *a priori* defined cognitive dysfunction, serum MMP-9 levels were higher in the following groups: (i) patients with NPSLE manifestations (73.2 ± 46.1 ng/mL) compared to patients without (42.8 ± 26.8 ng/mL); and (ii) patients with cognitive dysfunction (80.8 ± 45.8 ng/mL) compared to those without (48.6 ± 34.2 ng/mL) [64]. Further, serum MMP-9 levels correlated with volumes of T2-weighted lesions on brain MRI in SLE patients [64]. CSF MMP-9 levels were higher in SLE patients with NPSLE (240 ± 60 pg/mL) than in SLE patients without NPSLE (100 ± 20 pg/mL) and healthy controls (0.28 ± 0.16 pg/mL) [69]. CSF MMP-9 levels also correlated with CSF levels of tau and glial fibrillary acid protein, markers of neuronal degeneration and astrocytic degeneration respectively, in SLE patients [69]. Interestingly, CSF MMP-9 does not correlate with Q albumin but with CSF cell count, suggesting that CSF MMP-9 elevation seems to be a consequence of CSF cell invasion and accumulation [58]. The production of MMP-9 by different immune cells has to be taken into consideration. Granulocytes and monocytes are strong producers of MMP-9, whereas lymphocytes are weak producers, leading to different CSF MMP-9 levels depending on the CSF cytology [58]. In sum, these findings suggest a relationship between MMP-9 levels and SLE patients with neuropsychiatric involvement, and cognitive dysfunction in particular.

### 2.3. Neutrophil Extracellular Traps

Neutrophils act as the sentinels of the innate immune system, migrating from the circulating blood to sites of infection where they combat pathogens by phagocytosis, degranulation and the release of neutrophil extracellular traps (NETs) [70,71,72]. The discovery of NETs by Brinkmann in 2004 led to an entire new field of granulocyte investigation [71]. Activated neutrophils undergo “NETosis” to release NETs, a unique form of cell death that is distinct to necrosis and apoptosis [73,74]. NETs are fibrous structures consisting of chromatin backbones with diameters of ~17 nm with attached globular domains with diameters of ~50 nm [75,76]. The globular domains comprise of histones (H1, H2A, H2B, H3, and H4) and a granule-derived enzyme (neutrophil elastase, a neutrophil-specific serine protease) [71,75]. Other granule-derived enzymes present in NETs include myeloperoxidase and MMP-9 [71]. In total, less than 30 proteins are present in NETs and histones account for 70% of the protein mass [75,76]. The antimicrobial activity of histones is well established, killing bacteria at nanomolar concentrations more effectively than other antimicrobials [71,76]. NET formation has been documented in other conditions, including experimental models of neuroinflammatory diseases [77,78]. Transmigration across an activated endothelium *in vivo* is known to induce an increase in reactive oxygen species in neutrophils [78]. *In vitro*, transmigrated murine neutrophils (CD11b/Ly6G^+^) across IL-1β stimulated brain endothelium to undergo NETosis and caused cell death when applied to primary murine neuronal cultures [78]. Similar to the bacteria-killing experiments, addition of a monoclonal antibody against H2A-H2B-DNA complex improved neuronal viability [78]. A mixture of neutrophil proteases inhibitors (including inhibitor against neutrophil elastase) rescued neuronal viability when transmigrated neutrophils were applied to neurons in the presence of the protease inhibitors [78]. Interestingly, the protease inhibitors were ineffective when using conditioned medium from transmigrated neutrophils, suggesting that the cascade of events after the release of NETs might be neurotoxic [78]. *In vivo*, NETosis was observed in the cerebral cortex after sterotaxic intracerebral injection of LPS [78]. Taken together, the release of NETs containing histones and proteases that takes place normally as part of an antimicrobial defense potentially contributes to neurotoxicity [78]. This is in concordance with the observation that stroke patients with systemic infections have poorer outcomes [77,78].

Neutrophils from SLE patients (referred subsequently in text as lupus neutrophils) have altered functional properties, including diminished phagocytic capabilities, increased intravascular activation, increased platelet-neutrophil aggregation and increased production of reactive oxygen species compared to neutrophils from healthy controls (referred subsequently in text as control neutrophils) [79,80,81,82]. In addition, PBMC transcriptional signatures reveal overexpression of granulopoiesis-related genes in SLE patients but not in healthy controls [83]. This “granulocyte signature” is due to the presence of low-density granulocytes (LDGs) that co-purify with mononuclear cells during density centrifugation [83,84]. LDGs appear to be a distinct subset of immature neutrophils that includes a spectrum of pro-, myelo-, meta-myelocytes, early and late bands as well as some segmented neutrophils [82,83]. In addition, gene expression profiling of LDGs revealed an upregulation of mRNA of serine proteases (e.g., neutrophil elastase) present in primary, or azurophilic, granules [85]. Levels of mRNA expression that encode neutrophil serine proteases are greatest at the promyelocytic stage of neutrophil differentiation and are downregulated as the neutrophil matures [85]. The profile of cell surface markers of LDGs shows an increased expression of CD11b [86]. CD11b^+^ cells are believed to be pro-inflammatory and elevated numbers of CD11b^+^ cells correlate with disease activity in murine models of SLE [87]. Hence, LDGs appear to have a more immature and activated neutrophil phenotype compared to lupus neutrophils and control neutrophils [82]. Isolated LDGs demonstrate enhanced NET formation compared to lupus neutrophils and control neutrophils and are resistant to further NET induction (e.g., by phorbol 12-myristate 13-acetate), suggesting that LDGs may already be prestimulated *in vivo* [85,88]. NET degradation of SLE sera has been demonstrated to be impaired in a subset of SLE patients compared to healthy controls, particularly during disease flares [89,90]. Presence of deposited autoantibodies against NET components (e.g., anti-dsDNA and anti-histone) and complement (e.g., C1q) protect these NETs from DNase I digestion, allowing for the persistence of NETs in circulation [89,90]. This could be due to steric hindrance of the deposited autoantibodies and complement inhibiting DNase I access [90]. Tertiary, or gelantinase, granules of neutrophils contain MMP-9 [78,91]. Active MMP-9 is externalized on NETs at significantly higher levels in LDGs compared to lupus and control neutrophils [92]. Coculture of human umbilical vein endothelial cells (HUVECs) with human neutrophils induced to undergo NETosis leads to increased endothelial cell apoptosis [92]. This observation was completely abolished with the use of a transwell, indicating that direct contact between NETs and endothelial cells is required for cytotoxicity [92]. Furthermore, significant impairment of murine aortic endothelium-dependent vasorelaxation was observed in the presence of LDG-NETs [92]. This is mediated by MMP-9 on NETs activating endothelial matrix metalloproteinase-2 (MMP-2) to induce endothelial cell apoptosis and dysfunction [92]. Activation of endothelial MMP-2 was significantly decreased on exposure to MMP-9-depleted NETs compared to histone 2A-depleted NETs [92]. After adjusting for the concentration of NETs generated by control neutrophils and LDGs, vasorelaxation impairment induced by control NETs was similar to LDG-NETs; supporting the notion that it is the amount of MMP-9 released during NETosis that determines endothelial integrity in SLE [92]. Anti-MMP-9 antibodies are present in the sera of SLE patients and immune complexes containing MMP-9, but not irrelevant immune complexes (e.g., bovine serum albumin (BSA)/anti-BSA), enhanced NET formation via a FcγR signaling pathway [92]. Lastly, various spontaneous mouse models of SLE, in particular the MRL/MpJ-*fas*^lpr^ mice, demonstrate progressive cognitive impairment illustrated by defects in balance, learning ability and other cognitive impairments, temporally correlated with the onset of leukocyte trafficking into the cerebral microcirculation [93,94]. Increased leukocyte-endothelial cell interactions (leukocyte rolling and adhesion) have been observed in the pial microcirculation of MRL/MpJ-*fas*^lpr^ mice at 16 weeks of age compared to control MRL^+/+^ mice using intravital microscopy [93]. Further, analysis of the infiltrating leukocytes in brains of MRL/MpJ-*fas*^lpr^ mice at 16 weeks of age revealed mainly neutrophils as the dominant cell population present [94].

### 2.4. Pro-Inflammatory Mediators

Being an antigen-driven T cell-dependent B cell driven autoimmune condition, the effects of pro-inflammatory mediators in the context of NPSLE have been described [95]. In the lupus-prone (NZX × NZW)F1 (NZB/NZW) mice model, infiltration of CD3+ T cells and increased mRNA expression of pro-inflammatory mediators such as IL-1β, IL-6, IL-10, interferon (IFN)-γ and transforming growth factor β have been shown in the hippocampi *ex vivo* [95]. Further, these mice had evidence of memory impairment assessed using the novel-object recognition test [95]. Treating these mice with a tolerogenic peptide (human first complementarity-determining region) down-regulated the expression of the pro-inflammatory mediators and ameliorated the memory impairment in the mice [95]. B lymphocyte stimulator (BLyS) and a proliferation-inducing ligand (APRIL) are B cell stimulation and survival molecules [96]. CSF APRIL, but not BLyS levels, in 28 patients with NPSLE were significantly higher than those of disease controls with non-SLE related neurological disorders [96]. However, the levels of CSF APRIL and BLyS did not correlate with cognitive dysfunction in this study [96]. Cytokines are produced within the CNS by neurons and microglia, the surveillance cells of the CNS [97]. Studies have provided evidence of intrathecal production of IL-2, IL-6, IL-8 and IL10, which may be produced by the neuronal and micorglial cells [24]. BALB/c mice exposed to anti-NR2A/B antibodies and LPS had decreased metabolism on ^18^FDG microPET at the site of BBB breach, followed by a later increase in metabolic activity [98]. The initial decreased metabolism is presumably secondary to neuronal loss whereas the subsequent increase in metabolism may represent microglial inflammatory response to neuronal necrosis [98]. In human NPSLE, CSF IL-6 levels were associated with abnormal brain MRI signals which were mainly T2-weighted white matter hyperintensities [99]. Interestingly, CSF IL-6 correlated with CSF MMP-9 levels in 119 SLE patients, suggesting that BBB disruption might be involved in the development of brain MRI abnormalities in patients with NPSLE [69]. Depression, an important comorbidity of cognitive dysfunction, has been associated with significantly poorer cognitive function in patients with newly diagnosed SLE [48]. In an attempt to address whether panels of serum Th1 (IL-12, IFN-γ and tumor necrosis factor (TNF)-α) and Th2 (IL-5, IL-6, IL-10) cytokines were related to depression in 60 patients with childhood-onset SLE, only serum level of TNF-α was found to be associated with the level of depression in these patients, which is in keeping with the findings of a recent case-control study in patients with adult-onset SLE [100,101]. Hence, the impact of high serum TNF-α level on mood and, by conjecture, cognitive function appears to be substantial.

## 3. Conclusions

**Figure 1:**
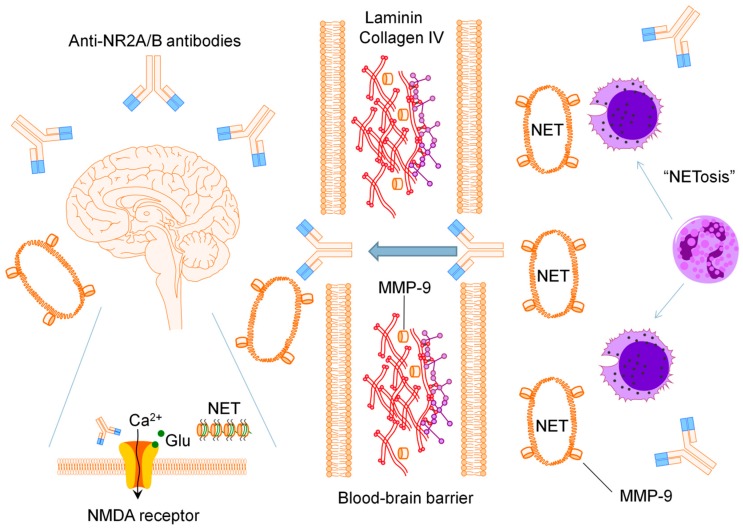
Proposed model of cognitive dysfunction in SLE. Abbreviations: NMDA, *N*-methyl-d-aspartate; MMP-9, matrix metalloproteinase-9; NET, neutrophil extracellular trap.

This review has focused on the potential pathogenic factors involved in cognitive dysfunction including anti-NR2A/B antibodies, MMP-9, NETs and pro-inflammatory mediators, based on the recent advances in cognitive dysfunction research from murine models and human SLE. Cognitive dysfunction in SLE sits at the interface of two of the most complicated organ systems in the human body, namely, the nervous system and the immune system. Obtaining clear and simple answers about disease causality is not easy, as evident from this review. In 1890, the great microbiologist Robert Koch established a set of postulates that have guided investigation into the pathogenesis of infectious diseases for over a century [102]. Koch’s postulates, which theorized that one microbe would cause one disease, cannot be applied for autoimmunity as the pathogenetic mechanisms leading to disease are multi-etiological in nature [102]. Based on the current information as discussed in this review, a hypothesis for the pathogenesis of cognitive dysfunction in SLE can be put forth as outlined in Figure 1. Anti-NR2A/B antibodies are produced extrathecally and must cross the BBB into the CSF to influence brain function. MMP-9 is released by prestimulated LDGs, leading to the degradation of basal lamina and compromise of the BBB integrity. Anti-NR2A/B antibodies activate the BBB with increased expression of endothelial cell adhesion molecules, facilitating the recruitment, rolling, adhesion and transmigration of neutrophils, leading to further NETosis intrathecally. Lastly, the intrathecal release of NETs contributes to neurotoxicity by inducing neuronal cell death, which subsequent leads to neuropsychiatric manifestations of SLE.

Neuropathology in this disease model requires two hits: (i) presence of neurotoxic anti-NR2A/B antibodies and (ii) a breach in the integrity of the BBB [37,103]. Thus, measurement of serum titers of anti-NR2A/B antibodies solely cannot be expected to correlate with antibody-mediated damage. The measurement of serum anti-NR2A/B antibodies, without CSF anti-NR2A/B antibodies levels or other biomarkers of BBB function, is therefore not ideal and likely the reason for the controversial results of the aforementioned 8 studies on anti-NR2A/B antibodies and cognitive dysfunction [9,19,31,43,44,45,46,47]. However, since obtaining CSF from SLE patients without overt neuropsychiatric manifestations is hindered by practical and ethical issues, non-invasive assessment of the integrity of the BBB opens an attractive research direction to study anti-NR2A/B antibody mediated cognitive dysfunction in SLE patients. Evident from this review, the process leading to cognitive dysfunction in SLE probably involves abnormal endothelial cell-immune cell interactions that permit access of immune cells (e.g., neutrophils) and therefore, immune mediators (e.g., NETs) into the CNS [16]. In mice models, the BBB has been compromised by simulating infection (e.g., lipopolysaccharide) and stress (e.g., epinephrine) [103,104]. However, such *in vivo* insults cannot explain the spontaneous development of BBB disruption and neuropsychiatric manifestations observed in anti-NR2A/B antibody positive MRL/MpJ-*fas*^lpr^ mice [97,105]. MMP-9 on NETs, released spontaneously by LDGs, might fulfill a critical role for the abrogation of BBB integrity in SLE. This is line with the recent findings of Brunner who screened biomarkers for the identification of cognitive dysfunction in childhood-onset SLE: (i) anti-NR2A/B antibodies; (ii) S100B (a biomarker of decreased BBB integrity); and (iii) neutrophil gelatinase associated lipocalin, S100A8 and S100A9 (all NET-reported proteomes) were included in the final predictive model with a sensitivity of 100%, specificity of 76% and area under ROC curve of 83.4% [31].

The discovery of anti-NR2A/B antibodies in mice mediating cognitive dysfunction represented a major advance in the field of cognitive dysfunction research; however, translating results from mice to men is fraught with difficulty, as illustrated in this review. Elucidating the pathogenesis of cognitive dysfunction and the origin of anti-NR2A/B antibodies in human SLE will require further basic investigation and a return to the laboratory is warranted to answer the remaining questions. Koch would have wanted it that way [102].
